# Metagenome-Assembled Genome of “*Candidatus* Aramenus sp. CH1” from the Chichon volcano, Mexico

**DOI:** 10.1128/mra.00526-24

**Published:** 2024-07-22

**Authors:** Roberto Marín-Paredes, Betsy A. Peña-Ocaña, Esperanza Martínez-Romero, Wilbert Gutiérrez-Sarmiento, Víctor Ruíz-Valdiviezo, Ricardo Jasso-Chávez, Luis E. Servín-Garcidueñas

**Affiliations:** 1 Laboratorio de Microbiómica, Escuela Nacional de Estudios Superiores Unidad Morelia, UNAM, Morelia, Michoacán, México; 2 Posgrado en Ciencias Biológicas, Unidad de Posgrado, UNAM, Ciudad de México, México; 3 Departamento de Bioquímica, Instituto Nacional de Cardiología Ignacio Chávez, Ciudad de México, México; 4 Tecnológico Nacional de México, Instituto Tecnológico de Tuxtla Gutiérrez, Tuxtla Gutiérrez, Chiapas, México; 5 Centro de Ciencias Genómicas, UNAM, Cuernavaca, Morelos, México; 6 Instituto de Neurobiología, Universidad Nacional Autónoma de México, Querétaro, México; Montana State University, Bozeman, Montana, USA

**Keywords:** *Sulfolobales*, archaea, metagenomics, volcano, crater lake, Chichon, extremophiles, Aramenus

## Abstract

The Chichon volcano contains several thermal manifestations including an acidic crater lake. Here we report a metagenome-assembled genome of “*Candidatus* Aramenus sp. CH1,” a *Sulfolobales* archaeon inhabiting the crater lake from the Chichon volcano. In this study, we generated a novel *Aramenus* genome sequence from a thermal area in Southern Mexico.

## ANNOUNCEMENT

The Chichon volcano (17.36°N, 93.23°W; 1,100 m above sea level) is located in southern Mexico and harbors a crater lake and hot springs with a temperature range of 35°C–82°C and a pH of 3.1–4.4 (1). The chemical composition of the volcanic sediments includes, principally, Al, Ca, Fe, K, Mg, Na, and Si (2). Recently, the presence of *Aramenus* archaea was reported at the Chichon Volcano (1). The *Aramenus* genus was first detected in the thermal field of Los Azufres in western Mexico (3). Here we report a metagenome-assembled genome (MAG) for an *Aramenus* species thriving in the crater lake of the Chichon volcano. The MAG was recovered to allow further comparative studies of *Aramenus* genomes from contrasting geographic sites.

A water sample from the crater lake of the Chichon volcano was collected on 8th November 2018, frozen in liquid nitrogen, and stored at −80°C. DNA was extracted with a QIAGEN DNA isolation kit (Hilden, Germany). DNA concentration was measured by NanoDrop One (Thermo Fisher, USA). Library preparation was carried out using a Nextera DNA Flex Prep kit following the manufacturer’s instructions (Illumina, USA). Sequencing was performed by an Illumina NextSeq platform 2 × 150 pair-ends reads at the Integrated Microbiome Resource, Halifax, Canada (1). Metagenomic reads were newly assessed using FastQC v.0.11.8 (4) and were filtered for quality (scores of ≥Q30) and adaptor sequences using Trim Galore v.0.6.7 (5). Reads were assembled *de novo* with SPAdes v.3.12.0 (6) using a combination of k-mer values (29, 37, 45, 53, 61, 79, 87, 95, 103, and 111). Binning was performed using DasTool v.1.1.2 (7) with the combination of Metabat v.2.12.1 (8), Maxbin v.2.2 (9), and Concoct v.1.1.0 (10). MAG contamination and quality were assessed using CheckM v.1.0.13 (11 – 13). Contigs were classified taxonomically using CAT v.5.2.3 (14). Scaffolding was performed using Swalo v.0.9.8-beta (15). Gap-filling was done using IMAGE2 v.2.4.1 (16). All programs were used with default settings. The metagenome was annotated using the Integrated Microbial Genomes & Microbiomes portal (IMG/M) (17, 18). The MAG was annotated using the NCBI Prokaryotic Genome Annotation Pipeline v.5.2 (19). The 16S rRNA sequence similarity, Average Nucleotide Identity calculation based on MUMmer (ANIm), and digital DNA-DNA hybridization (dDDH) values were calculated using BLAST v.2.13.0 local service (20), JspeciesWS (https://jspecies.ribohost.com/jspeciesws/#analyse) (21), and the Genome-to-Genome Distance Calculator v.3.0 (https://ggdc.dsmz.de/ggdc.php) (22), respectively.

The metagenome has 10,767,992 reads that were assembled into 3,561 contigs (*N*_50_ 111,558 bp) containing 5,194,736 bp. A 1,930,407 bp draft MAG comprising 24 contigs (*N*_50_ 224,069 bp), with a GC content of 48.86%, and genome coverage of 23× was obtained. The MAG has a completeness of 100%, with no detected contamination. MAG annotation predicted 2,243 genes.

Compared to the “*Ca*. Aramenus sulfurataquae AZ1” genome (ASRH00000000) from the thermal field of Los Azufres, 16S rRNA gene sequence similarity (98.39%), ANIm (92.82%), and dDDH (49.20%) values (23) indicate that the recovered MAG from the Chichon volcano may be considered a novel *Aramenus* species based on current genomic data.

This study allowed the recovery of a MAG corresponding to the archaeal genus *Aramenus* from the Chichon volcano. The recovered MAG may be useful for comparative genome analyses.

## Data Availability

The MAG, reads, and metagenome are available in GenBank, SRA, and JGI Genome Portal under accession numbers JALBWU000000000, SRR18166207, and 258897, respectively.
